# Beyond individual traits: differential associations of social support profiles with resilience in older learners

**DOI:** 10.3389/fpsyg.2026.1801980

**Published:** 2026-06-24

**Authors:** Qian Gao, Hui Zhou, Hang Lu

**Affiliations:** 1 School of Urban Management, Beijing Open University, Beijing, China; 2 Academic Affairs Office, Beijing Open University, Beijing, China; 3 School of Education, Zhejiang Normal University, Jinhua, Zhejiang, China

**Keywords:** active aging, K-means cluster analysis, older learners, resilience, social support systems

## Abstract

**Background:**

Lifelong learning is a vital pathway for fostering social integration and psychological well-being among older adults. This study examines the differential associations of social support and challenge patterns with resilience in older learners from a social-ecological perspective. Moving beyond the traditional focus on individual demographic traits, this study identifies multidimensional environmental factors—including family, school, and community support, as well as perceived learning barriers—to construct a Support-Challenge ecological analysis framework.

**Methods:**

K-means cluster analysis and robust Analysis of Variance (ANOVA) were employed to empirically analyze survey data collected from 1,783 older learners across 26 educational and residential learning sites in China.

**Results:**

Four distinct social support profiles among older learners were identified: Isolated-Comfortable, Balanced, Greenhouse-Empowered, and Supported-Challenged. Ecological configurations demonstrated significantly greater explanatory power for resilience than individual demographic characteristics. Notably, the Supported-Challenged group exhibited the highest levels of resilience, whereas the Isolated-Comfortable group demonstrated the lowest.

**Conclusion:**

These findings suggest that challenge and support synergistically influence the development of resilience in later life. Additionally, gender and education level serve as key factors predicting membership in different social support profiles. By integrating the dual dimensions of support and challenge, this study reveals the optimal ecological niche for resilience development, offering practical pathways for constructing empowering older adult learning ecosystems.

## 1 Introduction

As global demographic structures undergo irreversible shifts, the core agenda of gerontology has evolved from merely extending lifespan to optimizing healthspan and psychological adaptability. This study focuses on the Chinese context, which holds particular significance. As one of the countries with the largest elderly population and the fastest aging rate in the world, China is confronting unprecedented challenges in social security and public services (Liu et al., 2021; Yuan and Gao, 2020). In China, the latest data indicate that by the end of 2025, the population aged 60 and above reached 320 million, accounting for 23.0% of the total population (National Bureau of Statistics, 2026). In this context, exploring how to empower older adults through education to enhance their psychological resilience and quality of life is not only related to the well-being of hundreds of millions of Chinese elderly but also provides important regional experience for global strategies to address population aging (Tian et al., 2024; Formosa, 2019). Older adults face multifaceted challenges, including physiological decline, loss of social roles, and a widening digital divide, which increasingly highlight the vulnerability of their well-being (Fang et al., 2025). Against this backdrop, resilience is defined not merely as the capacity to recover from adversity but as a dynamic process of utilizing external resources for active adaptation and growth. It serves as a key internal resource for older adults to cope with aging-related challenges, achieve active aging, and sustain social participation (Färber and Rosendahl, 2020). Existing research indicates that the development of resilience is highly dependent on the social support systems in which individuals are embedded (MacLeod et al., 2016; Pechmann et al., 2014). Therefore, exploring how external supportive environments translate into the internal psychological capital of older adults has become an urgent public issue concerning active aging.

Lifelong learning is widely regarded as an external catalyst for enhancing resilience among older adults (Narushima et al., 2018). Theoretically, learning activities themselves provide older adults with opportunities to reconstruct social networks and acquire psychological resources (Lövdén et al., 2020). From a social-ecological perspective, learning in later life is not only a pathway for knowledge acquisition but also a process of constructing and maintaining multidimensional social support networks (Lee and Lee, 2024). As a microsystem, the learning environment integrates resources from schools, families, and communities (Sen et al., 2022). Multilevel social support provides older adults with avenues for coping with aging, cognitive activation, and social integration (Escolar and de Guzman, 2014). Empirical studies have also shown that older adults who participate in learning can establish broader social networks and demonstrate stronger psychological resilience in role transitions and life adaptation. Additionally, lifelong learning enables older adults to maintain occupational resilience and adapt to the changing labor market (Eppler-Hattab, 2021). Theoretically, a well-functioning learning support system can empower older adults, enabling them to transform external challenges into opportunities for resilience development when facing learning tasks and life stressors.

However, existing research has limitations in elucidating the relationship between support systems and resilience among older learners. Most studies tend to adopt a variable-centered approach, treating social support as a homogeneous linear variable or focusing solely on a single source of support (e.g., family only or peers only). This orientation overlooks the complexity and heterogeneity of social support systems. In reality, the support ecologies in which older learners are situated vary widely (Dumitrache et al., 2018). It remains unclear how these diverse sources of support resources and environmental challenges configure together to differentially shape the resilience of older learners. This research gap limits our ability to provide precise empirical evidence for enhancing the effectiveness of older adult education from the perspective of ecosystem design.

This study introduces a social-ecological perspective, shifting the focus to the social support ecological patterns of older learners. The study aims to move beyond the examination of isolated support variables to explore the relationship between specific combinations of multidimensional social support and challenges and resilience development. Specifically, this study focuses on the following core questions: First, is the explanatory power of traditional demographic characteristics on the resilience of older learners limited? Second, what unique social support system profiles do older learners form around the learning process? Third, what is the relationship between these heterogeneous support profiles and resilience levels and development? By systematically identifying the social support profiles of older learners and their association with resilience, this study aims to provide a solid empirical foundation for constructing an empowering and precise learning support ecosystem for older adults.

## 2 Literature review

### 2.1 Resilience

The term resilience originally referred to the ability of a stretched object to recover its size and shape after deformation, particularly deformation caused by compressive stress. Canadian ecologist Crawford Holling introduced the concept into the field of ecology, emphasizing a system’s capacity to absorb disturbance and reorganize for evolution rather than simple recovery (Holling, 1973). When extended to human society, it specifically refers to the capacity to recover from or adapt to significant trauma or rapid change. The American Psychological Association defines resilience as the process and outcome of adapting to difficult, challenging, and adverse situations. This process involves the ability to overcome difficulties, adapt to environments, and develop positive and flexible emotional and cognitive capabilities (Tsai and Freedland, 2022). Consequently, resilience encompasses the capacity to cope with both predictable and unpredictable life transitions and stressors, emphasizing a dynamic process of adaptation to adversity (Masten, 2014; Fleming and Ledogar, 2008). From a social-ecological perspective, resilience is not merely the ability to recover after “deformation” but a capacity for early warning and risk resistance based on learning, innovation, and adaptation (Walker, 2002).

Research on resilience spans individual, family, community, and national levels (Southwick et al., 2014; Chen et al., 2025). At the individual level, resilience is defined as the capacity to cope with life challenges, stress, and adversity (Waugh and Koster, 2015; Ye and Hu, 2024). However, recent perspectives view resilience through a more dynamic lens. It is characterized by dynamism, learnability, diversity, complexity, and engagement, reflecting the cross-system interaction between individuals and their social, built, and natural environments. An individual’s ability to adapt positively to unfavorable environments depends not only on personal traits but also on social and environmental factors, particularly the availability and responsiveness of services required to maintain well-being (Ungar, 2011). Therefore, the formation and development of resilience are influenced by multidimensional factors, including cognitive, emotional, behavioral, physiological, and environmental aspects (Ding et al., 2025). Many researchers note that resilience can be cultivated and is the result of the combined action of numerous internal and external protective factors (Garmezy et al., 1984). The most critical environmental factor is the social support network—namely, the breadth and depth of support provided by family, friends, and colleagues. The strength of a social support network is positively correlated with individual psychological resilience; individuals with robust support networks exhibit higher resilience when facing stress and adversity (Windle et al., 2011). Similarly, social support has been shown to enhance resilience in older adults (Wilks and Croom, 2008).

### 2.2 Older adult resilience

Older adult resilience emphasizes the ability of older adults to maintain health, adapt to changes in social roles, and sustain personal development when facing natural aging, environmental changes, or other crises. Older adult resilience encompasses multiple dimensions, including psychological aspects, physical function, and social relationships (Gooding et al., 2012; Jeste et al., 2013; Shen and Zeng, 2010). Specifically, Shen and Zeng (2010) developed a resilience scale designed for older adults based on Chinese health survey data, comprising dimensions such as self-acceptance, calmness, lack of loneliness, intimacy with family and friends, optimism, and life control. While traditional research focused on the static traits of resilience, the increasingly complex external contexts and diverse crises faced by older adults necessitate research grounded in real-world problem scenarios, emphasizing the ability to adapt, recover, and develop in the face of different stressors and challenges. For older adults, resilience emphasizes not merely survival and recovery, but the potential for adaptation and self-development.

Resilience holds significant protective potential for the overall health and quality of life of older adults (Färber and Rosendahl, 2020). It influences cognitive health and the aging process (Kalisch et al., 2015; Cohen et al., 2014). With higher psychological resilience, older adults are more willing to learn new skills, remain socially active, and engage in intellectually challenging tasks, all of which help maintain cognitive health. Furthermore, resilience influences life satisfaction (Gao et al., 2017; Ye and Zhang, 2021), helping older adults maintain subjective well-being and promoting successful aging (Ong et al., 2006). Older adults with high levels of resilience (e.g., emotional control, optimism, maintaining social relations, and independence) possess strong self-regulation abilities (Ashori and Aghaziarati, 2023). They are more adept at coping with adversities caused by physiological decline, shrinking social networks, or insufficient economic resources (Bonanno, 2004; Li et al., 2015). Consequently, resilience effectively mitigates negative emotions, improves lifestyles, enhances life satisfaction, and maintains overall well-being (Jeste et al., 2013; Hardy et al., 2004). Social support is a key environmental factor influencing older adult resilience. Numerous studies have confirmed that support from family, friends, and communities can significantly enhance older adults’ ability to cope with aging and adversity (Bielderman et al., 2015). In an environment with adequate social support, older adults can improve their social habits, establish positive interpersonal relationships, boost self-efficacy and self-esteem (Resnick, 2011), and enhance individual well-being (Lahar et al., 2023).

### 2.3 Older adult learning and resilience development

The diverse learning interests and needs of older learners provide significant opportunities for the development of educational services. Globally, the content of older adult education programs is becoming increasingly rich. Currently, learning activities can be categorized into several types: first, courses related to enhancing artistic appreciation and cultural literacy, such as painting, drama, and music; second, life skills and vocational training (Jenkins, 2011). Additionally, courses focused on memoir writing or autobiography creation attract older adults wishing to record life stories and memories (Boulton-Lewis, 2010). Furthermore, other programs relate to understanding the social environment beyond daily life, including cognition of social change and intergenerational interaction, such as global issue studies (Timmermann, 1998). As they age, older adults also need to master corresponding life skills and adapt to new technologies in social life, contingent upon changes in their physical and mental status and lifestyles.

The concept of lifelong education increasingly focuses on the human living environment and development, accounting for diversity and uncertainty. It posits that learning is crucial for coping with crisis and change, as it builds resilience and enhances sustainability (Wang, 2022). Older adult education must also address the specificity of the older adult lifeworld, aiming specifically to improve resilience in later life (Fu and Wu, 2023). Older adults who persist in learning can utilize their abilities to handle life difficulties effectively, accumulate learning experiences, cultivate cooperative and interactive learning awareness, establish extensive social networks, and form positive social experiences (Gibbons, 2003). Through learning and interaction at universities for the aged, older learners develop stronger psychological resilience and enhanced learning experiences. This enables them to actively adjust to dilemmas such as role maladjustment, relationship reconstruction, and cognitive decline, thereby adapting to new social environments and lifestyles, making their later life more fulfilling (Zhang, 2012).

### 2.4 Older adult resilience from the perspective of the social-ecological system

This study adopts the social-ecological system theory as its core analytical framework. This theory emphasizes that individual development does not occur in isolation but is embedded in a microsystem consisting of family and schools, a mesosystem formed by interactions among communities, families, and schools, and a macrosystem composed of sociocultural contexts and policies, resulting in individuals being nested within multi-level environments (Bronfenbrenner, 1979). For older learners, their resilience development is precisely the outcome of the interactive effects of these interconnected systems. Specifically, family, schools, and communities constitute the core microsystems influencing their learning and adaptation (Lamond et al., 2008). Supportive resources within these systems—such as emotional support, instructional guidance, and neighborhood mutual assistance—and challenging factors including learning difficulties and role transition pressures collectively shape older adults’ learning experiences and psychological adaptation processes (MacLeod et al., 2016). Based on this perspective, this study transcends the isolated examination of individual traits and shifts the unit of analysis from individual support variables to the configuration of support systems, aiming to reveal how the specific combinations of support and challenges in contextual settings differentially affect resilience.

From the core logic of the social-ecological system theory, the resilience of older learners is not solely determined by inherent individual traits but is a product of the dynamic interactions between micro, meso, and macro systems (Pechmann et al., 2014) At the micro level, family understanding and encouragement, professional instructional support from elderly education institutions, and the cultivation of a learning-oriented atmosphere in communities provide direct resource support for older adults. At the meso level, the collaborative cooperation between families and schools, as well as the resource linkage between communities and educational institutions, construct an interconnected network for resilience development. At the macro level, supportive aging policies and the popularization of lifelong learning concepts in sociocultural environments offer external guarantees for fostering resilience among older learners (Kalisch et al., 2015). This multi-system nested perspective breaks the dichotomous analytical logic of “individual-environment” in traditional research, better reflecting the complexity and contextuality of older adult resilience development, and provides a solid theoretical foundation for the subsequent identification of heterogeneous social support system profiles.

## 3 Materials and methods

### 3.1 Research instrument design

This study employed a cross-sectional design. The survey instrument comprised three sections. The first section collected demographic information. Based on the research objectives and existing literature, this study meticulously collected key demographic variables of the respondents, specifically including gender, age, highest educational level, and years since retirement. Furthermore, considering that factors such as health status, income level, marital status, chronic diseases, and digital literacy may potentially influence resilience, these variables were not included in the core measurement framework during the questionnaire design phase. In addition, this study controlled for extreme cases during the sample screening stage—for instance, excluding biases caused by severe cognitive impairment or acute illnesses to ensure the validity of research results—and these factors are explained as boundary conditions in subsequent discussions. This decision was based on the research focus but also constitutes a study limitation that requires careful consideration in subsequent discussions. These variables were included as predictors in the subsequent regression analyses.

The second section was the Older Adult Resilience Scale. Developed based on resilience theory and the active aging framework, this scale measures three dimensions of resilience: compensatory resilience, adaptive resilience, and developmental resilience. Compensatory resilience refers to the ability of older adults to cope with physical aging, maintain health, and compensate for losses (Freund and Baltes, 2002). Adaptive resilience denotes the capacity to adapt to role transitions and cope with changes in the social environment. Developmental resilience describes the ability to pursue self-development, enhance skills, and contribute to society. The scale demonstrated excellent reliability in the current sample (*N* = 1,783), with a Cronbach’s *α* coefficient of 0.957 for the total scale and coefficients exceeding 0.90 for all sub-dimensions. Confirmatory Factor Analysis (CFA) indicated a good model fit (*χ^2^/df* = 3.21, CFI = 0.956, TLI = 0.948, RMSEA = 0.045).

The third section involved the Older Adult Perceived Learning Support System Scale. Grounded in ecological systems theory, this scale assesses multi-level environmental factors subjectively perceived by older learners that influence their learning. The scale covers four dimensions: perceived family support, perceived school support, perceived community support, and perceived learning challenges or barriers. All items were rated on a 5-point Likert scale. Items related to learning barriers were reverse-coded prior to analysis to ensure consistent interpretation, where higher scores indicate higher levels of perceived support or lower levels of perceived barriers. In the present study, this scale also demonstrated good reliability, with a Cronbach’s α coefficient of 0.878 for the total scale and coefficients exceeding 0.80 for all sub-dimensions.

### 3.2 Data collection and analysis

Using a convenience sampling method, this study selected 4 universities for the aged, 17 community learning centers for older adults, and 5 nursing home learning centers as research sites. These three types of venues represent the primary settings where formal learning for older adults occurs. In selecting the research sites, this study balanced institutional scale, geographical location, and diversity of the student body: the universities for the aged cover both municipal and district levels, the community learning centers are distributed across different administrative regions of the city, and the nursing home learning centers include both public and private institutions—all to maximize the representativeness of the sample. Data were collected via electronic questionnaires, with uniformly trained research assistants providing on-site instructions and assistance. A total of 1,783 valid questionnaires were obtained. The sample comprised individuals aged 50–89 years, with women accounting for 80.4%, a figure that largely reflects the gender structure characteristics of current participants in older adult education, indicating a more active participation tendency among older women. Notably, the sample of this study is limited to urban older adults engaged in formal learning activities, and does not include rural older adults, older adults who do not participate in any learning programs, or groups unable to participate in learning due to severe health issues. This sampling scope was determined based on the research focus on older learners, but it also implies that caution is required when generalizing the conclusions.

Data analysis was conducted in several steps based on the research objectives. First, descriptive statistics and correlation analyses were performed to summarize basic sample characteristics and resilience profiles, and to examine intrinsic associations among the three resilience dimensions. Second, multiple linear regression analysis was conducted using demographic variables (e.g., gender, education, age, years since retirement) as predictors and total resilience scores and dimension scores as outcome variables. This analysis aims to address the first research question: whether the explanatory power of traditional demographic characteristics for resilience is limited. Prior to the regression analyses, all statistical assumptions (multicollinearity VIF < 3, normality of residuals, and Mahalanobis distance for outliers) were verified and met (see Supplementary Tables S1, S2, and Supplementary Figure S1).

To move beyond the examination of isolated variables and reveal holistic social support system types among older learners—addressing the second research question—K-Means cluster analysis was employed on 10 support and barrier variables. The validity and stability of the cluster analysis were cross-validated using the Silhouette coefficient, bootstrapped resampling (500 iterations), and split-sample replication. Subsequently, One-way Analysis of Variance (ANOVA) and the robust Games-Howell *post-hoc* test (chosen due to a violation of the homogeneity of variances assumption, as indicated by Levene’s test) were used to examine significant differences in resilience scores across profile types. Levene’s test revealed significant heterogeneity of variances across groups (*p* < 0.001), thus Games-Howell was employed for pairwise comparisons. This clarifies which ecological patterns are most conducive to resilience development, thus addressing the third research question: the relationship between heterogeneous support profiles and resilience levels. Finally, logistic regression analysis was utilized to explore which populations are more prone to falling into the highest-risk support system profiles, thereby identifying key risk factors.

## 4 Results

### 4.1 Descriptive statistics and limited explanatory power of demographic characteristics

The study sample comprised older adults aged 50–89 years, predominantly consisting of the young-old group (50–69 years, 84.6%), with a smaller proportion of the oldest-old (80 years and above, 4.3%). An analysis of resilience status revealed a generally high level of resilience among older learners (see Table 1). Notably, the oldest-old group (80–89 years) exhibited the highest total resilience score (4.41 ± 0.73), likely attributable to the survivor effect, where older adults capable of participating in learning activities inherently possess stronger adaptive capacities. Regarding gender, female learners accounted for 80.4% of the sample, significantly outnumbering males, and scored higher on resilience. This distribution aligns with the gender characteristics of participation in older adult education, reflecting a more active tendency toward social engagement among women. In terms of education, individuals with a bachelor’s degree or higher demonstrated the highest resilience levels (4.40 ± 0.80).

**Table 1:** Demographic characteristics of the sample (*N* = 1783).

Characteristic	Category	*n*	%	Resilience score (*M ± SD*)
Age	50–59	761	42.7	4.24 ± 1.14
	60–69	747	41.9	4.20 ± 1.11
	70–79	198	11.1	4.23 ± 0.99
	80–89	77	4.3	4.41 ± 0.73
Gender	Female	1,433	80.4	4.25 ± 1.07
	Male	350	19.6	4.16 ± 1.18
Education	Primary school or below	38	2.1	4.32 ± 1.23
	Junior high school	388	21.8	4.05 ± 1.26
	Senior high school/Vocational school	600	33.7	4.24 ± 1.10
	College (Associate degree)	415	23.3	4.23 ± 1.10
	Bachelor’s degree or above	342	19.2	4.40 ± 0.80

Regarding the three dimensions of resilience, adaptive resilience scored highest (*M* = 4.287), followed by compensatory resilience (*M* = 4.202), with developmental resilience being relatively lower (*M* = 4.200) (see Table 2). This distribution accords with the developmental trajectory of older adult learning, wherein older adults prioritize adapting to retirement life before pursuing self-development. The dimensions were highly correlated, with coefficients exceeding 0.92 (see Table 3). This indicates a high degree of synergy among compensatory, adaptive, and developmental resilience, suggesting that resilience in older learners acts as a dynamic, mutually reinforcing system rather than a simple aggregation of isolated dimensions.

**Table 2:** Descriptive statistics for resilience dimensions.

Dimension	*M*	*SD*
Compensatory resilience	4.202	1.120
Adaptive resilience	4.287	1.111
Developmental resilience	4.200	1.134
Total resilience	4.230	1.097

**Table 3:** Correlation matrix of resilience dimensions.

Dimension	1	2	3
1. Compensatory Resilience	—		
2. Adaptive Resilience	0.940^***^	—	
3. Developmental Resilience	0.937^***^	0.927^***^	—

***p < 0.001.

To investigate the impact of demographic variables (gender, education, age, years since retirement) on resilience, four multiple linear regression models were established with compensatory, adaptive, and developmental resilience, as well as the total resilience score, as dependent variables (see Table 4). Results indicated that education level had a stable but weak positive predictive effect on all resilience dimensions and the total score (*β*: 0.075–0.082). Men scored significantly lower than women on compensatory and developmental resilience, as well as the total score. However, the explanatory power (Adjusted *R*^2^) of all four models was extremely low (0.006–0.009), indicating that these demographic variables accounted for only approximately 1% of the variance in resilience.

**Table 4:** Regression analysis of demographic variables on resilience among older learners.

Predictor	Compensatory resilience		Adaptive resilience		Developmental resilience		Total resilience	
	β [95% CI]	*p*	β [95% CI]	*p*	β [95% CI]	*p*	β [95% CI]	*p*
Gender (Male = 1)	−0.150 [−0.275, −0.025]	0.019	−0.104 [−0.230, 0.022]	0.104	−0.133 [−0.259, −0.008]	0.037	−0.132 [−0.258, −0.006]	0.039
Education	0.082 [0.035, 0.130]	0.001	0.075 [0.028, 0.122]	0.002	0.080 [0.033, 0.127]	0.001	0.081 [0.034, 0.128]	0.001
Age	0.069 [−0.005, 0.143]	0.069	0.060 [−0.015, 0.134]	0.114	0.041 [−0.033, 0.116]	0.280	0.058 [−0.017, 0.132]	0.127
Retirement Years	−0.043 [−0.116, 0.030]	0.250	−0.042 [−0.115, 0.031]	0.256	−0.063 [−0.136, 0.010]	0.090	−0.050 [−0.123, 0.023]	0.174
Model stats								
*R^2^*	0.011		0.008		0.011		0.010	
*Adj. R^2^*	0.009		0.006		0.008		0.008	
*F*	4.96^***^		3.73^**^		4.77^***^		4.49^***^	

β = Standardized regression coefficient; CI, confidence interval. **p < 0.01, ***p < 0.001.

The multiple linear regression analysis revealed differential prediction patterns of demographic variables on resilience dimensions. Specifically, two statistically significant and stable patterns emerged. First, education level served as a positive protective factor across all dimensions. Despite the weak-to-moderate effect sizes, the consistency across dimensions suggests that educational background provides older individuals with richer cognitive resources, social capital, and problem-solving strategies to cope with external challenges. Second, gender emerged as a risk indicator for multiple dimensions. Compared to women, men exhibited significantly lower compensatory and developmental resilience and total resilience scores. Notably, among the predictors, gender (male) had the largest average absolute effect size, indicating that female older learners demonstrate greater psychological resilience, particularly in compensatory and developmental aspects.

Although all four regression models reached statistical significance (*F* values range: 3.73–4.96, all *p* < 0.01), the overall explanatory power was low. This suggests that the included demographic variables explain only a minimal fraction of the variance in resilience, implying that the primary sources of variation likely lie in social, behavioral, or environmental factors not included in these models. Thus, further investigation into how complex social-ecological systems play a decisive role is warranted.

### 4.2 Profile analysis of social support systems

**Figure 1:**
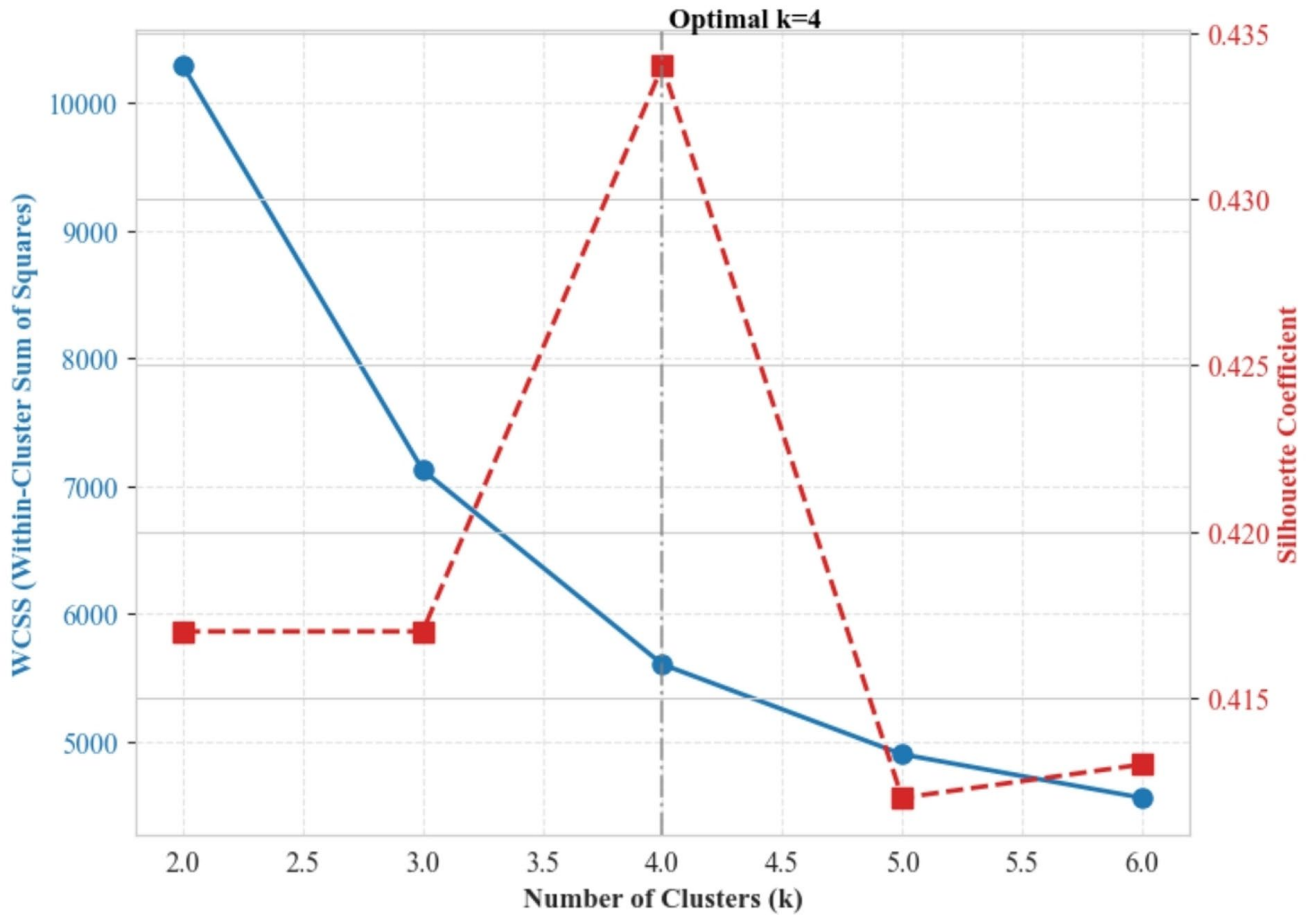
Determination of the optimal number of clusters using the elbow method (WCSS) and silhouette analysis.

To deeply reveal the environmental mechanisms influencing resilience, K-Means cluster analysis was conducted based on 10 support and barrier variables covering family, school, and community levels. Barrier variables were reverse-coded so that higher scores consistently represented higher support levels or lower perceived learning barriers (i.e., greater comfort). The optimal number of clusters was determined to be four based on the Elbow Method and Silhouette Coefficient (see Figure 1). Based on the overall patterns of support and barriers, the clusters were named as follows (see Table 5).

**Table 5:** Characteristics and distribution of the four support system profiles.

Profile	*n*	%	Family support	School support	Social support	Barriers (reverse)	Configuration
Isolated-comfortable	203	11.4%	1.53	1.57	1.47	4.55	Low support-low challenge
Balanced	455	25.5%	3.44	3.71	3.47	2.68	Mod. support-mod. challenge
Supported-challenged	777	43.6%	4.87	4.91	4.88	1.31	High support-high challenge
Greenhouse-empowered	348	19.5%	4.66	4.79	4.76	4.22	High support-low challenge

Bootstrapped stability analysis (500 resamples) yielded a mean Adjusted Rand Index (ARI) of 0.962, demonstrating exceptional statistical stability of the 4-profile structure. The 4-class solution also demonstrated good cluster separation, with a Calinski-Harabasz index of 1293.5 and a Davies-Bouldin index of 1.12 (see Supplementary Table S1). Furthermore, from a theoretical perspective, the 4-cluster solution was ultimately selected because a two- or three-cluster solution masked the critical distinction between the Greenhouse-Empowered (high support, low barriers) and Supported-Challenged (high support, high barriers) profiles. Distinguishing these diverse ecological environments represents the core theoretical focus of this study.

The four identified profiles are:

Isolated-comfortable: This group is characterized by extremely scarce social support but minimal perceived learning barriers (reverse score >4.5, indicating tasks are perceived as very simple). These older adults may be in a state of shallow learning lacking deep engagement and interpersonal interaction.

Supported-challenged: This is the largest group, possessing the highest levels of family, school, and community support, yet perceiving the greatest learning barriers (reverse score ~1.3, indicating significant difficulties). This suggests the group is tackling high-difficulty tasks while heavily relying on external support systems.

Greenhouse-empowered: This group enjoys high support and perceives low barriers, situated in an ideal environment with abundant resources and low pressure.

Balanced: This group shows moderate levels across all indicators, representing a relatively balanced state of moderate support and moderate barriers.

**Figure 2:**
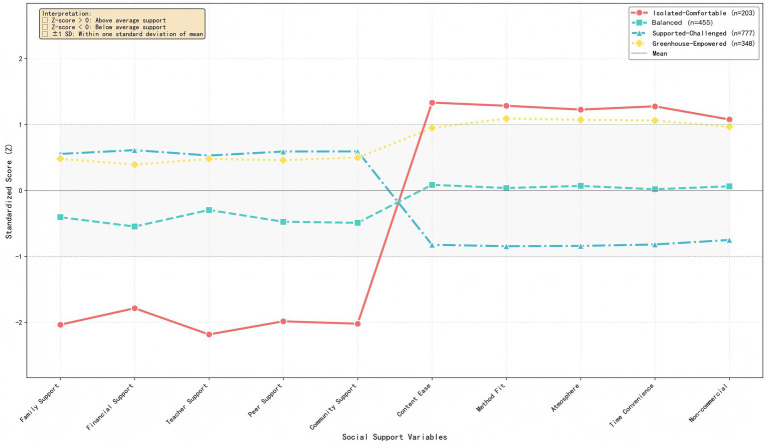
Profiles of social support systems among older learners.

As shown in Figure 2, the four profiles exhibit distinct configurations of support resources and perceived barriers. The Supported-Challenged and Isolated-Comfortable groups form an inverse crossover pattern. The Isolated-Comfortable group scores extremely low on support variables (left side, Z < −1.5) but extremely high on perceived ease (right side, Z > 1.0), vividly depicting an isolated state of comfort without support. Conversely, the Supported-Challenged group has the highest social support (left side, Z > 0.5) but the lowest scores on convenience/ease indicators (Z < −0.8), indicating they are coping with high-difficulty challenges bolstered by a strong support system. This stark divergence visually reveals the buffering role of social support in coping with learning challenges.

### 4.3 Differential associations of support system profiles with resilience

One-way ANOVA results indicated highly significant differences in total resilience and all dimension scores across the support system profiles (*p* < 0.001), with effect sizes (η^2^) ranging from 0.100 to 0.107, indicating moderate to large effects (see Table 6). Games-Howell *post-hoc* tests revealed that the Supported-Challenged group scored significantly highest on all resilience dimensions, followed by the Greenhouse-Empowered and Balanced groups, while the Isolated-Comfortable group scored significantly lowest. This result clearly demonstrates that a high-support, high-challenge environment is most conducive to cultivating psychological resilience in older learners, fully validating the critical role of social support systems.

**Table 6:** Comparison of resilience scores across support system profiles (*M ± SD*) and ANOVA results.

Profile	*n*	Compensatory resilience	Adaptive resilience	Developmental resilience	Total resilience
Isolated-comfortable	203	1.896 ± 0.613	1.984 ± 0.634	1.865 ± 0.616	1.915 ± 0.601
Balanced	455	3.805 ± 0.662	3.935 ± 0.661	3.799 ± 0.669	3.846 ± 0.647
Supported-challenged	777	4.821 ± 0.289	4.876 ± 0.279	4.831 ± 0.299	4.843 ± 0.273
Greenhouse-empowered	348	4.683 ± 0.366	4.773 ± 0.351	4.680 ± 0.382	4.712 ± 0.354
*F* value		1288.99^***^	1230.63^***^	1287.73^***^	1315.50^***^
*η* ^2^		0.103	0.107	0.100	0.106

***p < 0.001. Due to the heterogeneity of variances (Levene’s test *p* < 0.001), Games-Howell *post-hoc* tests were used for pairwise comparisons.

### 4.4 Analysis of demographic risk factors for the high-risk support system profile

To investigate the characteristics of high-risk populations, a binary logistic regression analysis was performed with the Isolated-Comfortable profile as the dependent variable (see Table 7). The results indicated that, after controlling for other variables, gender (male) (OR = 1.71, 95% CI [1.19, 2.44], *p* = 0.003) and lower education levels (OR = 0.73, 95% CI [0.64, 0.84], *p* < 0.001) were significant risk factors for falling into the “low support, low challenge” comfort trap. The negative predictive effect of age also reached statistical significance (OR = 0.78, *p* = 0.014), suggesting that relatively younger older learners may also face risks due to unestablished support networks. This further elucidates the pathway through which demographic variables operate: their influence does not act directly on resilience but rather indirectly contributes to lower resilience levels by increasing the probability of individuals falling into disadvantageous social-ecological systems.

**Table 7:** Logistic regression analysis of demographic risk factors for the isolated-comfortable profile.

Predictor	*B*	S.E.	Wald	*p*	OR [95% CI]
Intercept	−0.73	0.29	−2.51	0.012	–
Gender (male = 1)	0.54	0.18	2.93	0.003	1.71 [1.19, 2.44]
Education	−0.31	0.07	−4.31	<0.001	0.73 [0.64, 0.84]
Age	−0.25	0.10	−2.46	0.014	0.78 [0.63, 0.95]

Model Pseudo *R*^2^ = 0.023, LLR *p* < 0.001. OR, odds ratio; CI, confidence interval.

## 5 Discussion

By integrating traditional demographic analysis with innovative ecological profile analysis, this study systematically reveals the multiple mechanisms influencing the psychological resilience of older learners. The findings indicate that older adult resilience stems not merely from individual traits but relies more fundamentally on the overall quality and configuration of the social support systems in which individuals are embedded. This deepens the understanding of the essence of resilience in later life and provides an empirical basis for active aging practices to shift from focusing on individuals to designing ecosystems.

### 5.1 The multidimensional synergistic nature of resilience

The resilience of older learners is a multidimensional structure composed of compensatory, adaptive, and developmental capabilities that are intrinsically synergistic. Strong correlations were observed among these dimensions, reflecting the synergistic nature of resilience (Infurna, 2021). Older learners generally exhibit high levels of resilience (Gooding et al., 2012; Linnemann et al., 2020), concurrently mobilizing compensatory, adaptive, and developmental strategies when facing challenges. This synergy serves as a key mechanism for older adults to successfully adapt to change and adversity. Therefore, future initiatives could design comprehensive learning programs aimed at cultivating older adults’ multidimensional capacities for coping, integration, and growth.

### 5.2 The relationship between individual traits and resilience

Demographic variables serve as risk or protective factors influencing an individual’s entry into different social support profiles. Individual traits such as gender and education shape the initial social-ecological niche of the individual. This study found that educational background is the most stable, cross-dimensional individual protective factor predicting resilience in older adults. Early investment in education transforms into deep psychological resources for coping with aging challenges in later life—including efficient learning strategies, extensive social networks, and positive cognitive reframing (Perna et al., 2012; Hardy et al., 2004). Consequently, enhancing lifelong education levels for the entire population represents a strategic investment across the life course to hedge against the risks of an aging society.

Regarding gender differences, women demonstrated a resilience advantage (Perna et al., 2012). This likely stems from their ability to maintain relationships and build emotional support networks accumulated through long-term socialization, making it easier for them to create or benefit from environments where support and challenge coexist. Learning support services for older adults need to focus on social–emotional learning and the construction of support networks to compensate for potential deficits in relational resources among older men.

In addition to demographic variables, this study failed to statistically control for potential confounding factors such as health status, income level, marital status, chronic diseases, and digital literacy. These factors have been confirmed in existing research to be significantly correlated with older adults’ resilience and social support (Cornwell et al., 2008; Kim and Knight, 2014). For instance, better health status and higher digital literacy may enable older adults to access social support more easily, thereby influencing their resilience levels (Czaja et al., 2018); meanwhile, marital status and economic income may indirectly affect psychological resilience by shaping the size and quality of social networks. The low explanatory power of the regression models in this study (adjusted *R^2^ ≈ 1%*) may be partly attributed to the omission of these unmeasured variables. Future research should incorporate these factors into the model to more comprehensively reveal the complex relationships between individual characteristics and resilience.

### 5.3 An ecological system for resilience development with coexisting support and challenge

Based on the levels of social support received by older learners, this study identified four social support profiles. The analysis revealed that given adequate social support, challenges can promote the development of resilience. It is evident that resilience develops through the process of effectively coping with challenges. For older adults, when environmental support is sufficiently robust, it effectively buffers or even transforms the stress caused by high challenges. Supported by a strong system, the process of mobilizing resources, adapting, and overcoming difficulties serves precisely to synergistically reinforce compensatory, adaptive, and developmental resilience. Therefore, older adult education should encourage learners to step out of their comfort zones and gain genuine psychological growth from overcoming moderate challenges, provided that sufficient emotional and technical support is available. This finding echoes existing research on “challenging support”(Sanford, 1966) but further deepens our understanding of the synergistic effect of support and challenges. Previous studies have mostly focused on the direct positive effect of support on resilience (Seery et al., 2010; Rutter, 2012; Feeney and Collins, 2015; Crane and Searle, 2016), whereas this study reveals that the specific combination of support and challenges is the key determinant of resilience levels. The resilience of the high support-high challenge group is significantly superior to that of the high support-low challenge group. This indicates that not all high-support environments can maximize resilience development; moderate challenges are a necessary condition for achieving a leap in resilience.

Social support promotes the development of resilience (Chang and Yarnal, 2018). The Isolated-Comfortable group exhibited the lowest level of resilience, indicating that the absence of support from family, school, and community weakens the adaptive and developmental capacities of older learners (Gooch et al., 2016; Chu, 2010). Although this group perceives fewer pressures and barriers, the lack of necessary social support and connection fails to stimulate individual coping and growth motivations. Thus, the core task in promoting older adult resilience is to help individuals build or enter an ecosystem abundant in supportive resources and establish a sense of belonging (Bennett et al., 2023).

### 5.4 Promoting equity in support systems

This study found that the oldest-old group (80 years and above) had the highest total resilience scores in the descriptive statistics, which corroborates the finding that the Supported-Challenged profile exhibits the strongest resilience. On one hand, older adults who can persist in learning into advanced age possess inherent advantages in health and motivation; on the other hand, they possess robust and efficient social support systems that assist them in coping with aging-related challenges. Support systems not only facilitate the development of current resilience but also serve as a lifeline for maintaining long-term quality of life and psychological adaptation. Future governance in aging societies should focus not merely on eliminating barriers caused by aging but on translating the supportive ecology enjoyed by these survivors into an accessible and scalable public support system, particularly to benefit vulnerable older adults unable to participate in formal learning due to health or socioeconomic reasons.

From the theoretical perspective of social ecology, this study shifts the focus of resilience research from “what individual traits older adults possess” to “what ecological configurations individuals are embedded in” by identifying four heterogeneous support profiles. This shift expands the analytical level of resilience theory, indicating that resilience is not only a reflection of psychological capabilities but also an outcome of the interaction pattern between individuals and their environment. The identification of different profile types provides an empirical foundation for understanding the multiple pathways of resilience development and lays a theoretical basis for designing hierarchical and targeted intervention strategies.

### 5.5 Limitations and future directions

This study utilized a cross-sectional design; thus, causal inferences should be interpreted with caution. Regarding sample representativeness, this study has the following main limitations: First, the sample is only derived from urban older adults participating in formal learning, and does not cover rural older adults, older adults who do not participate in any learning programs, or groups unable to participate in learning due to severe health or functional impairments. Although this sampling scope helps focus on the research question, it also limits the generalizability of the conclusions. Second, the sample has a high proportion of females (80.4%), which, while largely consistent with the actual gender structure of participants in older adult education, may lead to insufficient observation of the resilience formation mechanisms of male learners. Future research should attempt to include a broader sample, including rural older learners, male learners, and older adults with different health statuses, to test the cross-group stability of the research conclusions.

In terms of variable control, this study failed to incorporate potential confounding variables such as health status, income level, marital status, chronic diseases, and digital literacy into the model for statistical control. These factors may both affect older adults’ access to social support and their resilience levels, and their omission may have a certain impact on the accuracy of the results. Although this study reveals the holistic characteristics of support system profiles through cluster analysis, which partially offsets the reliance on individual confounding variables, future research should still include these variables in future research designs to more accurately estimate the net effect of each factor on resilience.

In addition, future research can adopt a longitudinal design to track the dynamic covariation trajectory of support system profiles and resilience, and further explore the key influencing factors of profile type transformation. At the same time, combining qualitative interviews can more vividly reveal the specific life narratives and psychological processes of older adults constructing resilience and mobilizing resources under different social support profiles.

Finally, while grounded in ecological systems theory, this study primarily operationalized selected levels (microsystem and mesosystem: family, school, community). Several broader ecological levels, such as the exosystem, macrosystem (e.g., social policies, cultural values), and chronosystem, were not included. Future research should incorporate these broader levels to provide a fully comprehensive ecological analysis.

## 6 Conclusion

Resilience in older learners is a malleable, multidimensional, and synergistic dynamic capability system. Its development is highly dependent on the configuration of the social support ecosystem. The holistic ecological pattern constituted by multiple supports and challenges from family, school, and community is key to predicting and explaining differences in resilience. Therefore, the fundamental path to promoting resilience in older adults should shift from focusing solely on individual ability training to the meticulous design and precise intervention of social support ecosystems.

Based on the findings, it is recommended to build a precision-stratified ecosystem for resilience promotion. First, support systems should be solidified by focusing on training professional teachers for older adult education and establishing peer mentorship programs. Community-embedded learning sites should be promoted to encourage older adults to learn locally. Simultaneously, family co-learning programs should be encouraged to extend support from the individual to the family unit. Second, the potential of advantaged groups should be unleashed by providing specialized, high-level courses for high-resilience groups, transforming them from learners into contributors. Finally, for medium- and low-resilience groups, basic and supportive services should be strengthened. Learning programs designed with higher sociability, practicality, and lower cognitive thresholds should be developed, prioritizing the construction of early success experiences and peer connections to compensate for potential disadvantages in relational resources or cognitive strategies.

## Data Availability

The original contributions presented in the study are included in the article/supplementary material, further inquiries can be directed to the corresponding author.
