# Supplementary material for: Beyond individual traits: differential associations of social support profiles with resilience in older learners
Source: Front Psychol. 2026 Jun 24;17:1801980. doi: 10.3389/fpsyg.2026.1801980 (PMC13341973; doi:10.3389/fpsyg.2026.1801980)
Supplement: Supplementary file 1 [file Table_1.DOCX]

**Table S1.** Clustering Validation Summary

|  | Metric | Value |
| --- | --- | --- |
| 1 | Silhouette Coefficient | 0.434 |
| 2 | Calinski-Harabasz Index | 1293.51 |
| 3 | Davies-Bouldin Index | 1.122 |
| 4 | Bootstrap ARI (Mean, 500 resamples) | 0.962 |
| 5 | Bootstrap ARI (Standard Deviation) | 0.023 |
| 6 | Split-sample Silhouette Difference | 0.014 |
| 7 | Split-sample Center Distance | 0.286 |
| 8 | Outliers (Mahalanobis distance) | 0.84% (n=15) |

**Table S2.** Multicollinearity and Homoscedasticity Diagnostics

| Assumption Test | Variable / Dimension | Statistic | Conclusion |
| --- | --- | --- | --- |
| Multicollinearity (VIF) | Gender | 1.07 | No multicollinearity |
|  | Education | 1.00 | No multicollinearity |
|  | Age | 1.06 | No multicollinearity |
| Homoscedasticity (Levene's test) | Compensatory Resilience | F=149.32, p<.001 | Assumption violated* |
|  | Adaptive Resilience | F=186.65, p<.001 | Assumption violated* |
|  | Developmental Resilience | F=135.81, p<.001 | Assumption violated* |

Note. Because the assumption of homogeneity of variances was violated (*p*<.001), the robust Games-Howell post-hoc test was utilized for all pairwise group comparisons.

**Figure S1.** Q-Q Plot of Pearson Residuals for Logistic Regression


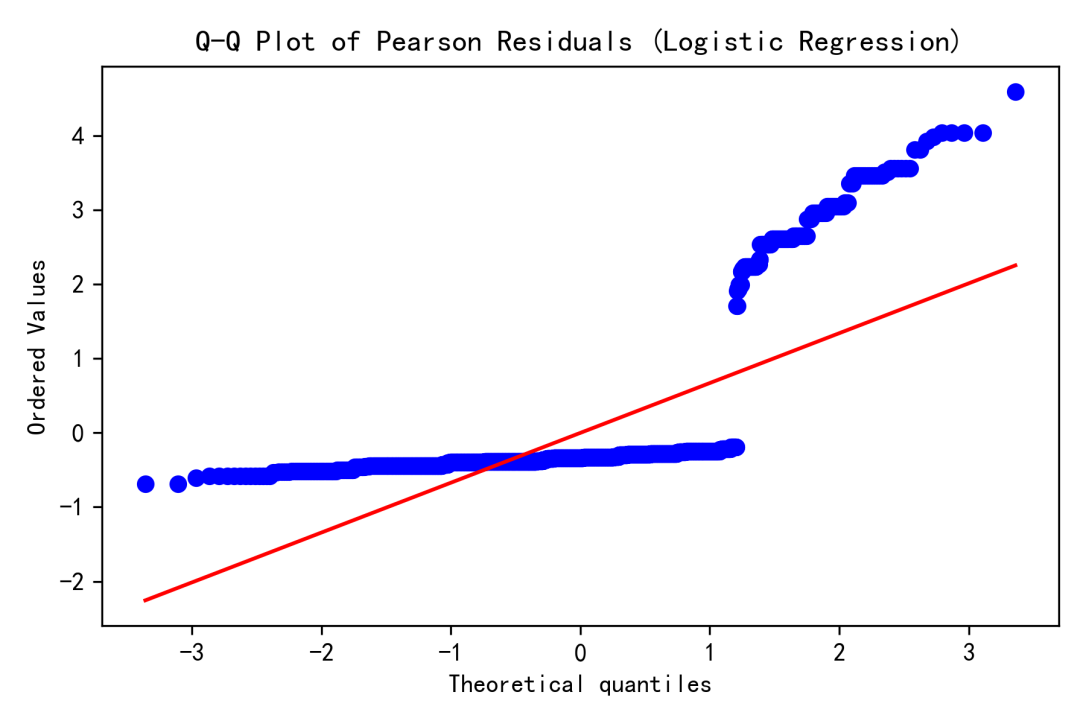


Note. The Q-Q plot shows that residuals approximately follow the normal distribution, supporting the validity of the logistic regression model.
